# Identification and characterization of a novel gene controlling floral organ number in rice (*Oryza sativa* L.)

**DOI:** 10.1371/journal.pone.0280022

**Published:** 2023-01-05

**Authors:** Phyu Phyu Maung, Backki Kim, Zhuo Jin, Su Jang, Yoon Kyung Lee, Hee-Jong Koh

**Affiliations:** Department of Agriculture, Forestry and Bioresources, Plant Genomics and Breeding Institute, Research Institute for Agriculture and Life Sciences, Seoul National University, Seoul, Korea; China National Rice Research Institute, CHINA

## Abstract

Floral organ number is crucial for successful seed setting and mature grain development. Although some genes and signaling pathways controlling floral organ number have been studied, the underlying mechanism is complicated and requires further investigation. In this study, a floral organ number mutant was generated by the ethyl methanesulfonate treatment of the Korean *japonica* rice cultivar Ilpum. In the floral organ number mutant, 37% of the spikelets showed an increase in the number of floral organs, especially stamens and pistils. Histological analysis revealed that the number of ovaries was determined by the number of stigmas; spikelets with two or three stigmas contained only one ovary, whereas spikelets with four stigmas possessed two ovaries. The floral organ number mutant showed pleiotropic phenotypes including multiple grains, early flowering, short plant height, and reduced tiller number compared with the wild-type. Genetic and MutMap analyses revealed that floral organ number is controlled by a single recessive gene located between the 8.0 and 20.0 Mb region on chromosome 8. Calculation of SNP-index confirmed *Os08g0299000* as the candidate gene regulating floral organ number, which was designated as *FLORAL ORGAN NUMBER7* (*FON7*). A single nucleotide polymorphism (G to A) was discovered at the intron splicing donor site of *FON7*, which caused the skipping of the entire sixth exon in the mutant, resulting in the deletion of 144 bp. Furthermore, the T-DNA-tagged line displayed the same floral organ number phenotype as the *fon7* mutant. These results provide valuable insight into the mechanism of floral organ differentiation and formation in rice.

## Introduction

The floral organs of spikelets, especially stamens and pistils, are fundamentally important for the fertilization process, and control normal seed setting and mature grain development [1]. Homeotic transformation of floral organs alters the floral organ number or leads to the complete loss of floral organs, causing abnormal seed formation or no seed setting in rice [2]. Therefore, understanding the factors affecting floral organ number at the molecular level is crucial for normal seed development and for gaining insight into yield improvement.

A milestone understanding of the detailed molecular mechanism and the ABC model of floral organ patterning was first developed in eudicots, namely, *Arabidopsis thaliana* and *Anthirrhinum majus* [3]. Subsequently, the regulation of D- and E-class genes in floral organ development was identified, and the ABC model was updated to the ABCDE model [4]. In rice, a modified version of the ABCDE model was established based on the identification of numerous genes regulating floral and spikelet development [4–9]. In this model, the A-class genes (*OsMADS14*, *OsMADS15*, *OsMADS18*, and *OsMADS20*) regulate the formation of lemma and palea [7]; B-class genes (*OsMADS2*, *OsMADS4*, and *OsMADS16*) and C-class genes (*OsMADS3*) regulate stamen identity [4, 10]; C-class genes (*OsMADS58*) determine pistil identity [11]; D-class genes (*OsMADS13*, and *OsMADS21*) regulate the specification of the ovule development [12, 13]; E-class genes (*OsMADS1-LHS1*, *OsMADS5*, *OsMADS6*, *OsMADS7*, *OsMADS8*, and *OsMADS34*) specify the identities of stamens, pistils, and ovary [10, 13, 14].

Floral organ specification is determined not only by the ABCDE model, but also by the CLAVATA (CLV)–WUSHEL (WUS) signal transduction pathway. Mutations in *CLV* genes in *Arabidopsis* cause the progressive enlargement of floral meristem, resulting in flowers with extra sepals, petals, stamens, and ovaries [15]. The CLV–WUS module regulates floral organ number through the negative regulation of stem cell accumulation and positive regulation of floral meristem [16]. Rice *FLORAL ORGAN NUMBER1* (*FON1*) and *FON2/FON4* genes encode *Arabidopsis* CLV1 and CLV3 homologs, respectively [17–19]. Thus, the FON1–FON2 signaling pathway in rice corresponds to the CLV1–CLV3 signaling system in *Arabidopsis*. Additionally, the rice CLV–WUS pathway has been proven to regulate floral meristem and floral organ number in other crop plants, such as Maize, Brassica, Tomato [16]. The *FON2/FON4* genes act in parallel with other floral homeotic regulators such as, *OsMADS16*, *OsMADS58*, *OsMADS13*, and *OsMADS1* [20]. FON1 is required for the activation of FON2/FON4 protein function, whereas the other CLV3/EMBRYO SURROUNDING REGION (CLE) homolog, FON2 SPARE1 (FOS1), can substitute for FON2 activity without requiring FON1 [21]. Two other CLE homologs, FON2-LIKE CLE PROTEIN1 (*FCP1*) and *FCP2*, negatively regulate vegetative stem cell activity and promote leaf initiation by repressing the expression of *WUSCHEL-RELATED HOMEOBOX4* (*WOX4*) [16, 22].

In addition, *SUPERWOMAN1 (SPW1*), *DROOPING LEAF* (*DL*), *abnormal floral organ (afo)*, *TONGARI BOUSHI1 (TOB1)/YABBY5*, retrotransposon *Tos17*, *osmads1-z*, *ABERRANT PANICLE ORGANIZATION 1* (*APO1*, ortholog of *UFO*), and *ABERRANT PANICLE ORGANIZATION 2* (*APO2*, ortholog of *LFY*) also control floral organ identification in rice [14, 23–27]. Recently, studies on the interaction among *FON4*, *APO1*, and C- and D-class genes suggested a regulatory module that fine-tunes floret patterning and floral organ determinacy in rice [10]. Thus, accumulating evidence indicates that floral organ development is a multi-step process and involves numerous genes in a spatiotemporally regulated manner [4]. Despite these findings, our understanding of the floral organ regulatory pathway remains limited. Identification of additional floral organ genes is required to attain a clear understanding of the molecular mechanism of floral organ and spikelet development.

In this study, we characterized a chemically mutagenized *japonica* rice mutant exhibiting increased floral organ (stamen and pistil) numbers. The gene underlining floral organ number (*FON7*) was identified by MutMap analysis, and its function was confirmed using a T-DNA-tagged line.

## Materials and methods

### Plant materials

The *fon7* mutant was generated by the ethyl methanesulfonate (EMS) treatment of the Korean *japonica* rice cultivar Ilpum. F_1_ and F_2_ populations, derived from crosses between the mutant and wild-type rice, were used for the genetic analysis and mapping of *FON7*. All plants were grown under normal conditions in the experimental paddy field of the Seoul National University, Suwon, Korea.

### Agronomic and morphological characterization of the mutant

The agronomic traits of wild-type and mutant plants, including plant height, tiller number, panicle length, and internode length, were measured in seven biological replicates, and statistically analyzed using SPSS version 25. During flowering, five panicles from each of five wild-type and mutant plants were randomly selected, and the components of each floret were investigated and photographed under a microscope using HD’MEASURE (HANA Vision, Incheon, Korea).

### Histological analysis

The freshly collected spikelets were fixed in formalin-acetic acid-alcohol (FAA; 5% formaldehyde, 5% acetic acid, and 45% ethanol), and stored at 4°C. The fixed spikelets were dehydrated in a graded ethanol series from 65% to 100%. Then, the samples were infiltrated with xylene substitute for 2 h, dipped in paraffin for 3 h, and subsequently embedded in new paraffin. The paraffin-embedded samples were sectioned into 8 μm-thick slices using the MICROM HM 325 Rotary microtome (Microm Lab, Germany). These sections were deparaffinized in xylene, stained with 0.05% toluidine blue, and photographed under the Olympus CX 31 light microscope (Olympus, Japan).

### Genetic analysis of the *fon7* mutant

A total of 179 F_2_ plants derived from crosses between mutant and wild-type (Ilpum) plants were subjected to genetic analysis; F_2_ plants exhibiting increased floral organ numbers, short plant height, early flowering, and multiple grains were considered as mutants. The segregation ratios of F_2_ populations were analyzed by chi-square (χ^2^) test in SPSS version 25.

### Identification of the *floral organ number* gene using MutMap

DNA was extracted from the young and healthy leaf samples of wild-type and F_2_ mutant plants using the cetyltrimethylammonium bromide (CTAB) method. The genomic DNA samples of 12 F_2_ mutant plants were combined in equal amounts, and the bulked DNA was sequenced on the Illumina NovaSeq platform at the National Instrumentation Center for Environmental Management (NICEM) of Seoul National University (NICEM, Seoul, Korea). The resequencing data of Ilpum (Illumina Hiseq 2500) was used for MutMap analysis (version 2.3.2). Subsequently, the SNP-index plot was generated using SNPs with a minimum SNP-index of 0.4 within 3 Mb windows.

### SNP genotyping using derived cleaved amplified polymorphic sequence (dCAPS) markers

Candidate SNPs with SNP-index > 0.9 were selected as potential causal SNPs responsible for the changes in floral organ number. The dCAPS Finder 2.0 (http://helix.wustl.edu/dcaps/dcaps.html) was used to design dCAPS markers for the selected SNPs (S5 Table). The PCR products were digested with appropriate restriction enzymes for 2 h, and analyzed by electrophoresis on 3% agarose gel. The sizes of wild-type and mutant PCR products were visualized under the ImageQuant LAS 4000 biomolecular imager (GE Healthcare Bio-Sciences Corp, USA).

### Identification of exon skipping in *fon7* mutant

To identify nucleotide changes and splicing patterns in the coding region of the candidate gene, total RNA was isolated from wild-type and mutant leaves using the GeneAll Hybrid-R^™^ kit (GENEALL Bio, South Korea), and then treated with RNase-free Recombinant DNase I (Takara Bio, Japan) to eliminate genomic DNA contamination. Then, cDNA was synthesized from total RNA using the M-MLV reverse transcriptase kit (Promega, Madison, WI, USA), and amplified using candidate gene-specific primers. PCR products were purified using the DNA purification kit (Inclone, Korea), and subjected to Sanger sequencing with both forward and reverse primers. Nucleotide changes were detected by aligning the wild-type and mutant cDNA sequences using the Codon Code Aligner software (Codon Code Corporation, USA).

### RNA extraction and qRT-PCR analysis

Spikelets and seeds were sampled from the *fon7* mutant plants and the T-DNA-tagged line as well as from the corresponding wild-types (Ilpum and Dongjin, respectively) in three biological replicates, with each replicate containing three technical repeats. Total RNA was extracted from the harvested plant samples using the TakaRa MiniBEST Plant RNA Extraction Kit (TaKaRa Bio, Kusatsu, Japan), and first-strand cDNA synthesis was carried out using oligo (dT) primers and M-MLV reverse transcriptase (Promega, Madison, WI, USA). Quantitative real-time PCR (qRT-PCR) was performed using sequence-specific primers and SYBR Premix ExTaq (TaKaRa, Japan) on the CFX96 Real-Time PCR System (Bio-Rad, Hercules, CA, USA), according to the manufacturer’s instructions. Expression levels of *FON7* were normalized relative to those of *Actin*, a housekeeping gene, and relative gene expression levels were calculated using the ΔΔCt method.

### Validation of the mutation responsible for altered floral organ number

The function of *FON7* gene was validated using the T-DNA-tagged line provided by KyungHee University. PCR-based genotyping of T-DNA insertion mutant plants was conducted using the T-DNA-specific left border primer in combination with gene-specific primers. Then, homozygous T-DNA insertion mutants were selected by loading the PCR products on 1% agarose gel. The phenotypic traits and relative *fon7* expression levels of homozygous T-DNA mutants were analyzed and compared with those of the wild-type.

### Multiple sequence alignment

The MEMO1 protein sequences of some plant species were downloaded from the Gramene database (https://ensembl.gramene.org/index.html). Then, multiple sequence alignment of these amino acid sequences was conducted using Codon Code Aligner (version 6.0.2.6; Codon Code, Centerville, MA, USA). Identical amino acids were shaded, and their percent identity with rice MEMO1 was calculated.

## Results

### Phenotypic characterization of the *fon7* mutant

The *fon7* mutant was identified from the EMS-mutagenized library of the Korean *japonica* rice cultivar Ilpum. Weak vigor and short plant height of the *fon7* mutant were apparent at the seedling stage. Additionally, the mutant flowered approximately two weeks earlier than the wild-type (Table 1), and showed significant reduction in plant height, internode length, panicle length, tiller number per plant, and spikelet number per panicle compared with the wild-type (Table 1 and S1 Fig).

**Table 1:** Agronomic traits of the wild-type and *fon7* mutant.

Genotype	Traits^a^						
	Normal florets (%)	Abnormal florets (%)	No. of days to heading	Plant height (cm)	Tiller number	Panicle length (cm)	No. of spikelets per panicle
**Wild-type**	100	0	108	100.1±3.8	11.4±2.6	23.1±1.2	131.1±8.2
***fon7***	62.98	37.02	95	75.6±2.9**	8.4±0.8**	19.5±1.1**	89.9±9.1**

^a^Data represent mean±standard deviation (SD; n = 7). Asterisks indicate significant differences (**P < 0.01; unpaired *t*-test).

### Floral organ morphogenesis of the *fon7* mutant

**Fig 1:**
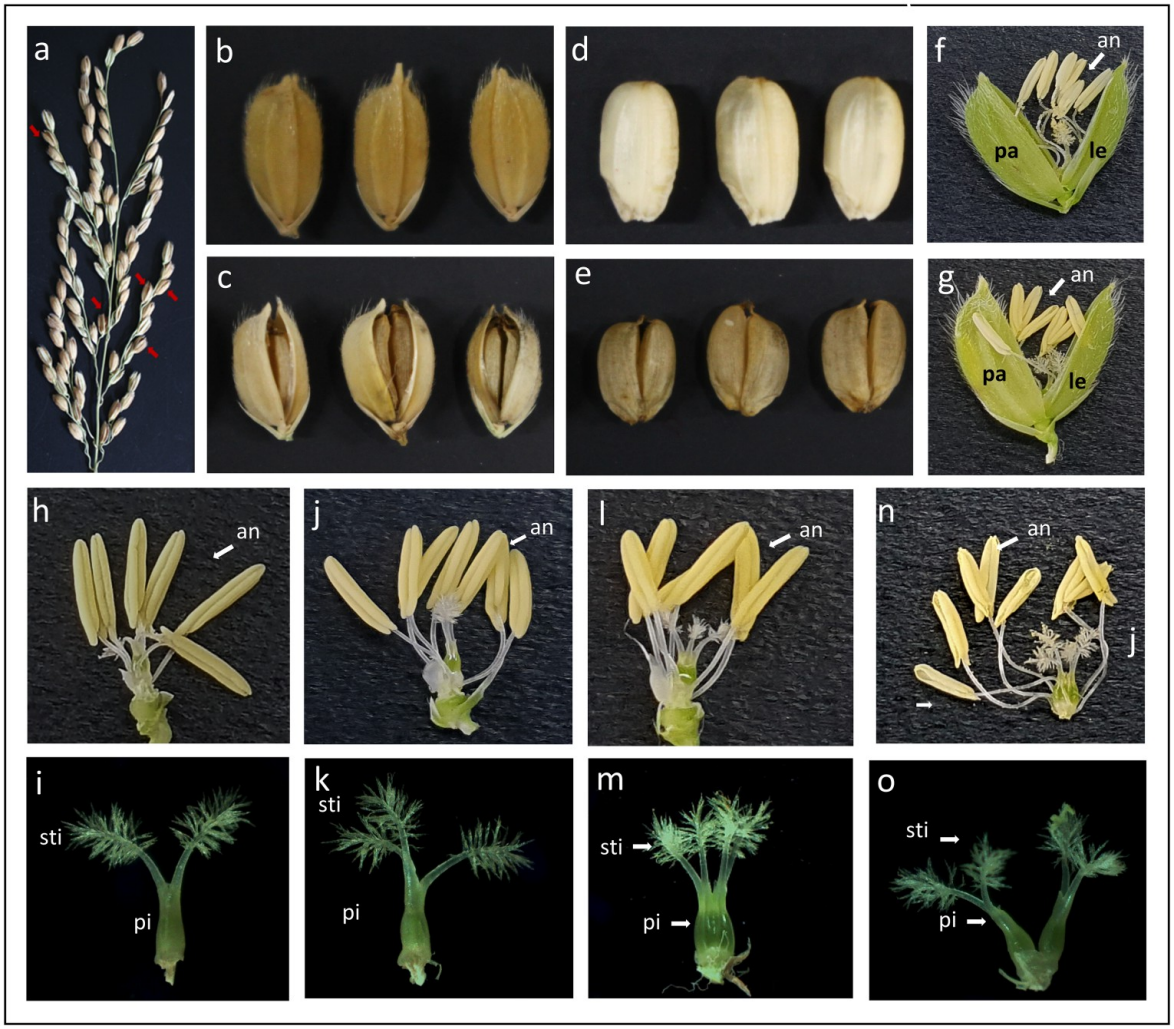
Grain and floral organ phenotypes of the wild-type and *fon7* mutant. (a) Occurrence of multiple grains (red arrows) in the *fon7* mutant. (b, c) Wild-type (b) and *fon7* mutant (c) grains. (d, e) Wild-type (d) and *fon7* mutant (e) grains with hull removed. (f, g) Wild-type (f) and *fon7* mutant (g) spikelets. (h) Wild-type spikelet with lemma and palea removed. Six stamens and two stigmas are visible. (i) Wild-type pistil with two stigmas. (j) *fon7* spikelet with lemma and palea removed, showing seven stamens and three stigmas. (k) *fon7* pistil with three stigmas. (l) *fon7* spikelet with lemma and palea removed, showing six stamens and four stigmas. (m) *fon7* pistil containing four stigmas, with two ovaries attached to each other. (n) *fon7* spikelet with lemma and palea removed, showing eight stamens and four stigmas. (o) *fon7* pistil containing four stigmas, with two ovaries separated from each other. All spikelets in five panicles of five randomly selected plants of each genotype were examined, and representative images are shown. an, anther; pi, pistil; sti, stigma.

The *fon7* mutant showed the multiple-grain phenotype (Fig 1a), whereby a single set of lemmas and palea enclosed two seeds, each of which could germinate and develop into a whole plant (S2 Fig). The wild-type plants produced only normal grains, whereas the *fon7* mutant plants produce both normal and multiple grains (Fig 1b–1e).

**Fig 2:**
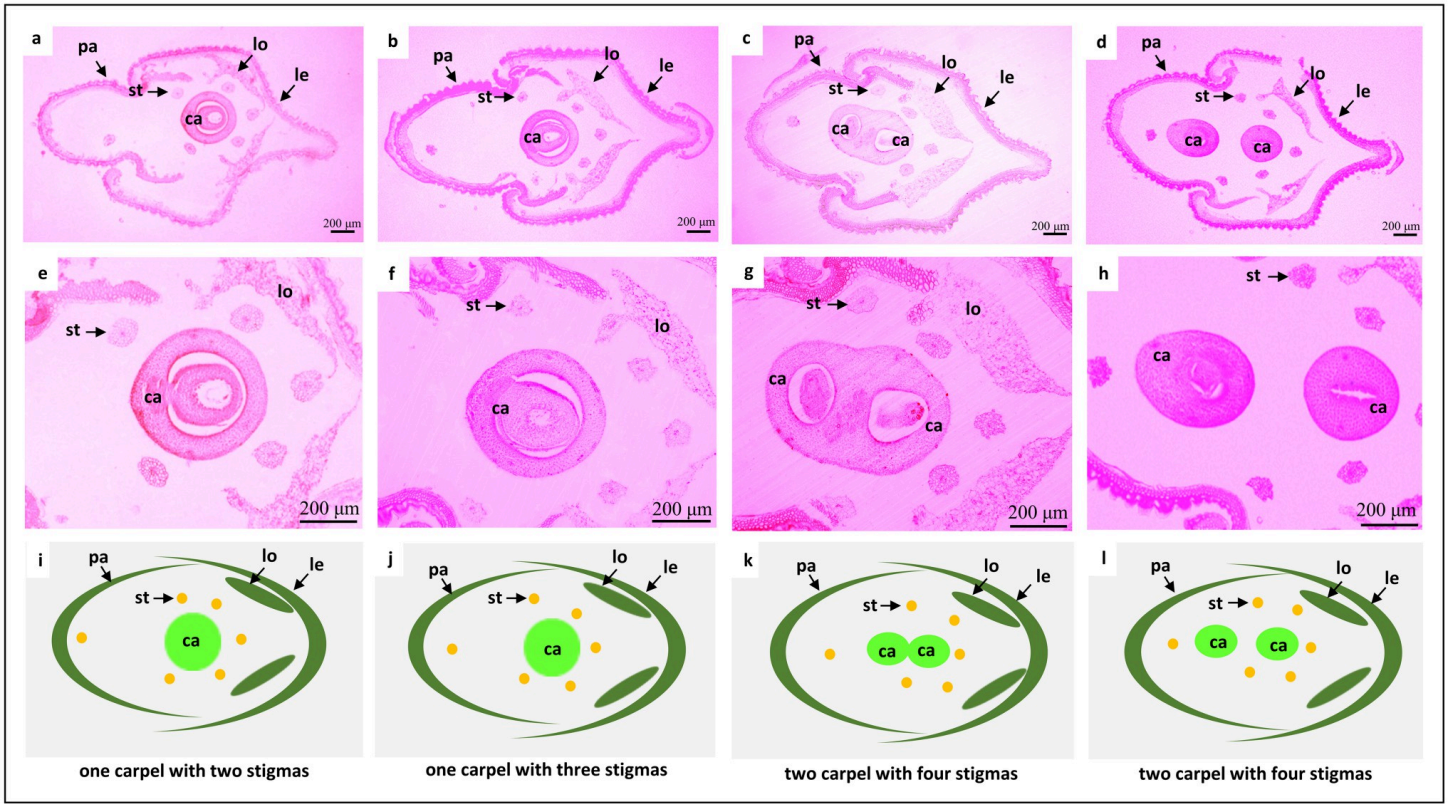
Microscopic analyses of paraffin sections of wild-type and *fon7* mutant spikelets located at the basal position of the corresponding spikelets. (a) Transverse section of wild-type spikelet. (b–d) Transverse sections of *fon7* spikelets formed from flowers with three stigmas and one ovary (b), four stigmas and two fused ovaries (c), and four stigmas and two separated ovaries (d). (e–h) Transverse sections of the ovary in upper spikelets. (i–l) Sketches depicting the upper photos of paraffin sections. ca, carpel; le, lemma; lo, lodicule; ov, ovary; pa, palea; st, stamen.

A normal rice floret is composed of one pistil (that produces one ovary), six stamens, two lodicules, one palea, and one lemma (Fig 1f). While the *fon7* mutant spikelets showed no obvious differences in the outer whorl floral organ number (lemma, palea, and lodicules) compared with the wild-type, they exhibited an increased number of stamens and pistils (Fig 1g). All florets in the wild-type showed normal florets containing six stamens and two stigmas (Table 1, Fig 1h and 1i). However, 37% of florets in the *fon7* mutant exhibited abnormal florets with increasing floral organ number, i.e., up to nine stamens or up to two pistils and four stigmas (Table 1 and Fig 1j–1o). The correlation between stigma number and ovary number per spikelet was investigated through histological analysis of the transverse section of wild-type and mutant spikelets. Interestingly, normal spikelets with two stigmas or abnormal spikelets with three stigmas contained only one ovary, while those with four stigmas showed two ovaries (Fig 2a–2h). The two ovaries contained within a spikelet were sometimes attached and sometimes located separately (Fig 2g and 2h). Schematic representations of the transverse sections of wild-type and mutant spikelets are presented in Fig 2i–2l.

### Genetic analysis of the *fon7* mutant

The F_1_ and F_2_ progenies derived from crosses between the wild-type (Ilpum) and mutant plants were phenotypically evaluated at the heading stage. All F_1_ plants showed wild-type phenotypes, suggesting that the floral organ number mutant trait was recessive. In the F_2_ population, 137 out of a total of 179 plants exhibited wild-type phenotypes; however, phenotypes of the remaining 42 plants showed the *fon7* mutant traits including increased stamen and pistil numbers, early flowering, short plant height, and multiple grains. Additionally, the chi-square test revealed the wild-type: mutant segregation ratio of 3:1 (χ ^2^ 0.225 < χ^2^_0.05(1)_ = 3.841), indicating that floral organ number is controlled by a single recessive gene.

### Identification of the gene controlling floral organ number in rice

**Fig 3:**
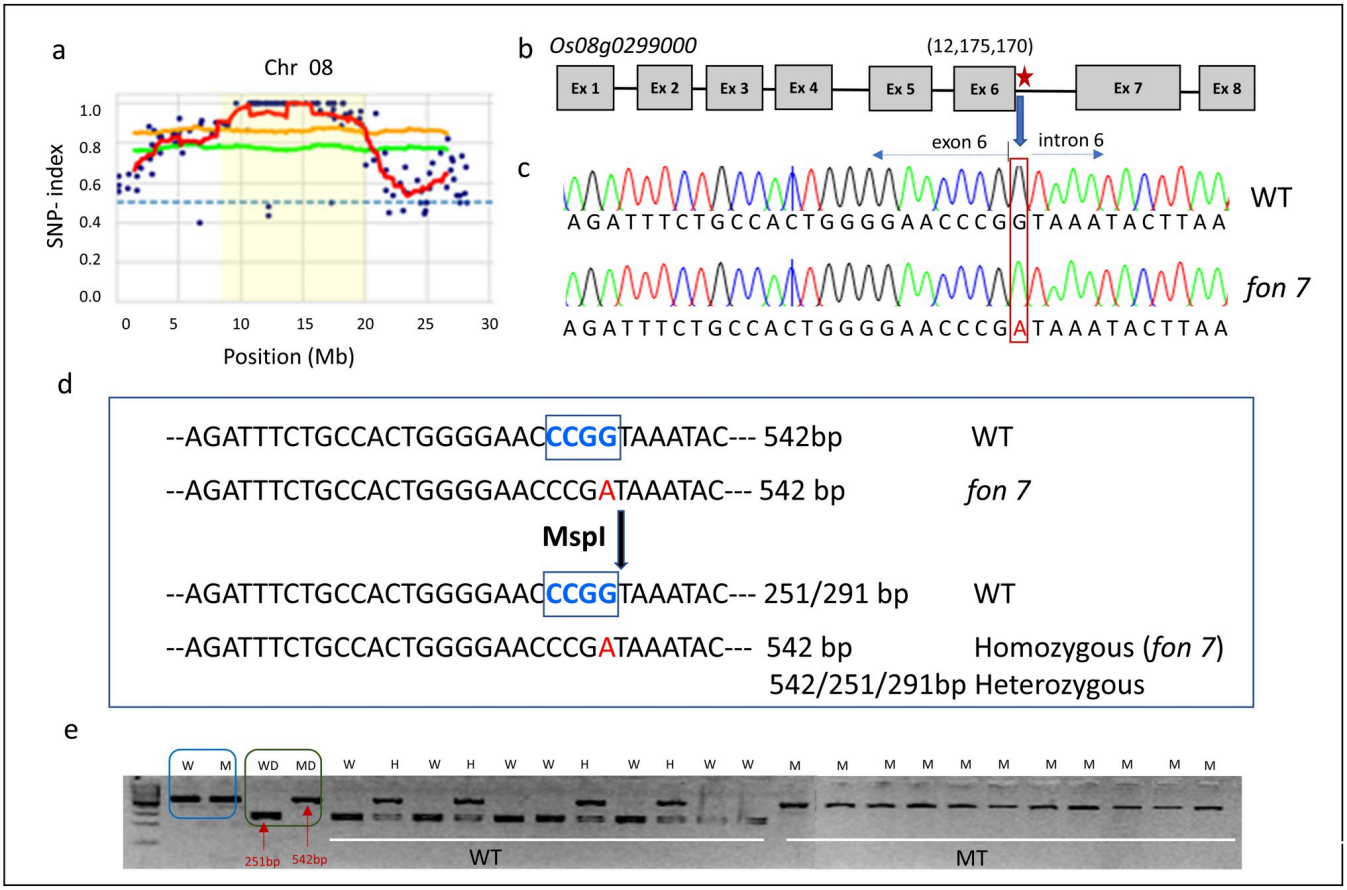
SNP-index graph and development of dCAPs marker. (a) SNP-index graph generated by the MutMap analysis of the *fon7* gene. Green and orange lines indicate 95% and 99% confidence intervals, respectively. Red regression lines were obtained by averaging SNP indices from a moving window of five consecutive SNPs and shifting the window one SNP at a time. Blue dots represent SNP-index values at the SNP position. Y-axis shows SNP-index values ranging from 0–1, and X-axis indicates the SNP position (Mb). Pale-yellow shaded area on chromosome 8 indicates the region corresponding to the candidate gene controlling floral organ number in rice. (b) Gene structure of *Os08g0299000*. Gray boxes represent exons; black lines represent introns; red star indicates the SNP position. (c) Sequence comparison between the wild-type and *fon7* mutant. Red color indicates the SNP (G→A). (d) Development of the dCAPS marker for *fon7*. The sequence within the gray box indicates the mismatched base, which was used for developing the dCAPS marker. (e) Co-segregation of the SNP with the floral organ number phenotype of F_2_ plants derived from the *fon7* mutant × wild-type (Ilpum) cross. PCR products were digested with *Msp*I, and then separated on agarose gel. WT, wild-type; MT, mutant; WD, wild-type DNA fragment digested with *Msp*I; MD, mutant DNA fragment digested with *Msp*I.

Causal candidate SNPs responsible for the changes in floral organ number were predicted using MutMap analysis. SNP plots were generated by calculating the SNP-index of each SNP from the Illumina data of bulked F_2_ mutant DNA. Among all 12 chromosomes of rice, the average SNP peak index was detected in the 8–20 Mb region of chromosome 8, which was selected as the candidate region (Fig 3a). A total of 97 SNPs and indels were detected in the candidate regions of chromosome 8 (S3 Fig, S3 and S4 Tables). Among these polymorphisms, three SNPs designated as SNP-1 (*Os08g0223833*; a frameshift variant), SNP-2, (*Os08g0299000*; a splice donor variant), and SNP-3 (*Os08g0408200*; a missense variant) with SNP-index > 0.9 were found in coding regions or at a splice site (S4 Table). SNP-2 (*Os08g0299000*), a G-to-A polymorphism at the 12,175,170 bp position, was used to develop dCAPS markers (Fig 3b–3d). Genotyping the F_2_ population using SNP-2 dCAPS marker revealed a complete co-segregation with altered floral organ number phenotype, thus confirming that *Os08g0299000* controls the changes in floral organ number in rice (Fig 3e).

To clarify whether the SNP-2 in *Os08g0299000* is naturally found in the rice germplasm, the nucleotide sequence flanking the 12,175,170 bp position on chromosome 8 was compared among the sequencing data of 4,726 cultivated rice accessions available at the RiceVarMap v2.0 website. No SNP was detected near this region in rice accessions, suggesting that the SNP responsible for the *fon7* mutant phenotype does not represent natural variation in the rice germplasm (data not shown). Therefore, we identified *Os08g0299000* as the candidate gene controlling floral organ number in rice.

**Fig 4:**
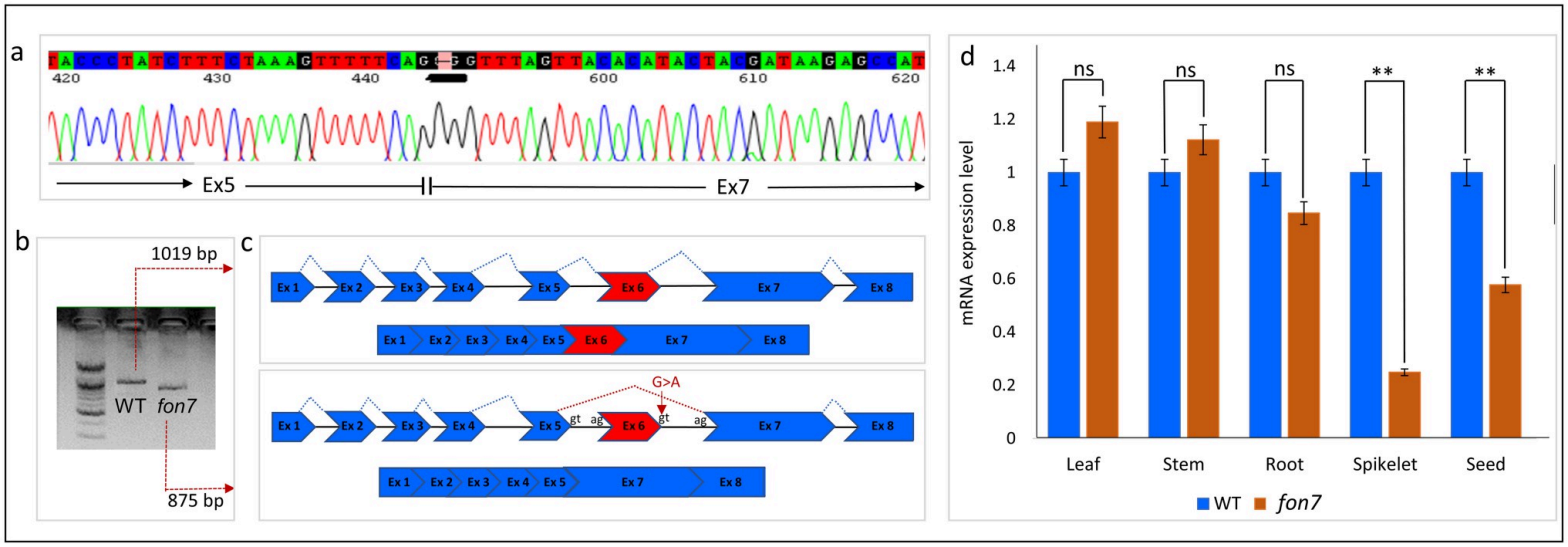
Exon skipping of *fon7* mutant. (a) cDNA sequence analysis of the *fon7* locus in the mutant. (b) Gel electrophoresis of RT-PCR products of the *FON7* gene in the wild-type and *fon 7* mutant. (c) Gene structure of *Os08g0299000* in the wild-type and mutant. Blue boxes represent exons, and gray lines represent introns. The mutation occurred in intron 6, changing G to A (splice donor variant), which caused the deletion of exon 6 (exon skipping). (d) Relative expression level of *Os08g0299000* in the wild-type (WT; Ilpum) and *fon7* mutant as examined by real-time quantitative PCR (qRT-PCR). Asterisks indicate significant differences (**P < 0.01; unpaired *t*-test). ns, non-significant.

Because the G-to-A SNP in the intron 6 (splice donor site) of *FON7* was associated with increasing floral organ number (stamens and stigmas) in rice, we determined the full-length *FON7* cDNA sequence in the wild-type and *fon7* mutant to examine potential differences in the deduced amino acid sequence of FON7 between the two genotypes. The full-length *FON7* cDNA sequence was 1,019 bp in the wild-type, as predicted, but was shorter in length in the *fon7* mutant (Fig 4a and 4b). Sanger sequencing revealed that *FON7* cDNA carried a 144 bp deletion in the *fon7* mutant compared with the wild-type. Sequence comparison revealed that full-length *FON7* cDNA contains eight exons in the wild-type but lacks the entire exon 6 in the mutant (Fig 4c). Consequently, the FON7 protein is predicted to contain 298 amino acids in the wild-type but only 250 amino acids in the mutant. Overall, this result suggests that G-to -A mutation at the intron donor site of *FON7* fails to splice and leads to exon skipping in the *fon7* mutant.

### Expression analysis of *FON7*

The spatial expression pattern of *FON7* was determined by qRT-PCR to further understand the gene function. The *fon7* mutant showed significantly lower expression levels of *FON7* in the reproductive tissues, especially spikelets and seeds, than the wild-type (Fig 4d).

### Validation of the mutation causing altered floral organ number

**Fig 5:**
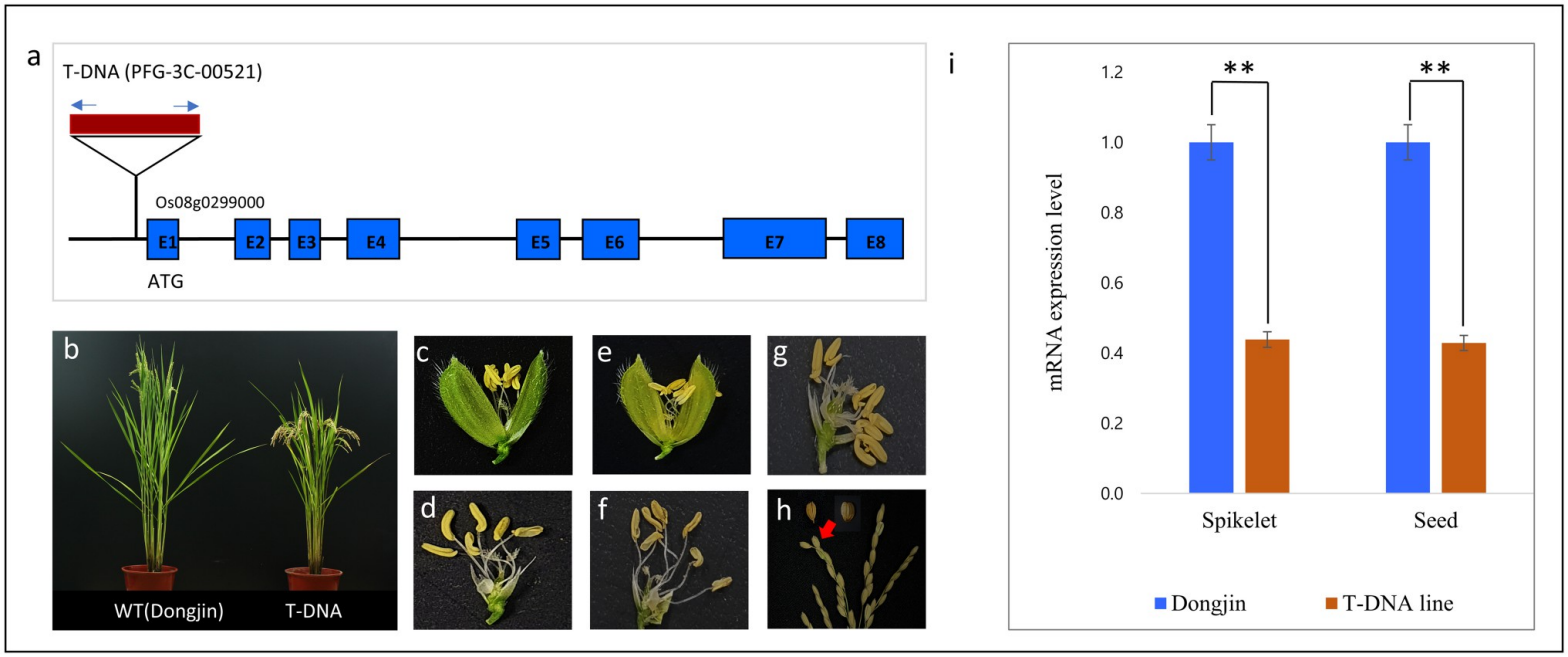
Morphological and *FON7* expression phenotypes of the T-DNA-tagged line. (a) T-DNA insertion position in the *FON7* gene model. (b) Comparison of plant morphology between the wild-type and T-DNA-tagged line. (c) Wild-type spikelet. (d) Wild-type spikelet with lemma and palea removed, showing six stamens and two stigmas. (e) Spikelet of the T-DNA plant. (f, g) Spikelet of the T-DNA plant with lemma and palea removed, showing seven stamens and two stigmas (f) or eight stamens and four stigmas (g). (h) Occurrence of multiple grains in the T-DNA plant. WT, wild-type (Dongjin); T-DNA plant, PFG_3C-00521. Red arrowheads indicate seeds containing multiple grains. (i) Relative expression level of *Os08g0299000* in the T-DNA plants and its corresponding wild-type (Dongjin), as examined by qRT-PCR. Asterisks indicate significant differences (**P < 0.01; unpaired *t*-test). ns, non-significant.

The role of *Os08g0299000* in floral organ number determination was verified using a T-DNA-tagged line (PFG_3C-00521). T-DNA was inserted in the promoter region of the *Os08g0299000* gene (Fig 5a). Homozygous T-DNA-tagged line exhibited early flowering and short plant height compared with the wild-type (Fig 5b); these traits of the homozygous T-DNA insertion mutants were consistent with those of the *fon7* mutant. In addition, all wild-type florets were phenotypically normal; however, florets produced by the homozygous T-DNA tagged line exhibited increased floral organ number (stamens and pistils) and multiple grains (Fig 5c–5h). These results indicated that the mutant phenotype is caused by the loss-of-function mutation of *FON7*. Compared with the wild-type, the relative expression levels of *FON7* were significantly lower in the spikelet and seed of the homozygous T-DNA tagged line (Fig 5i).

### Homology analysis of the MEMO1 protein

**Fig 6:**
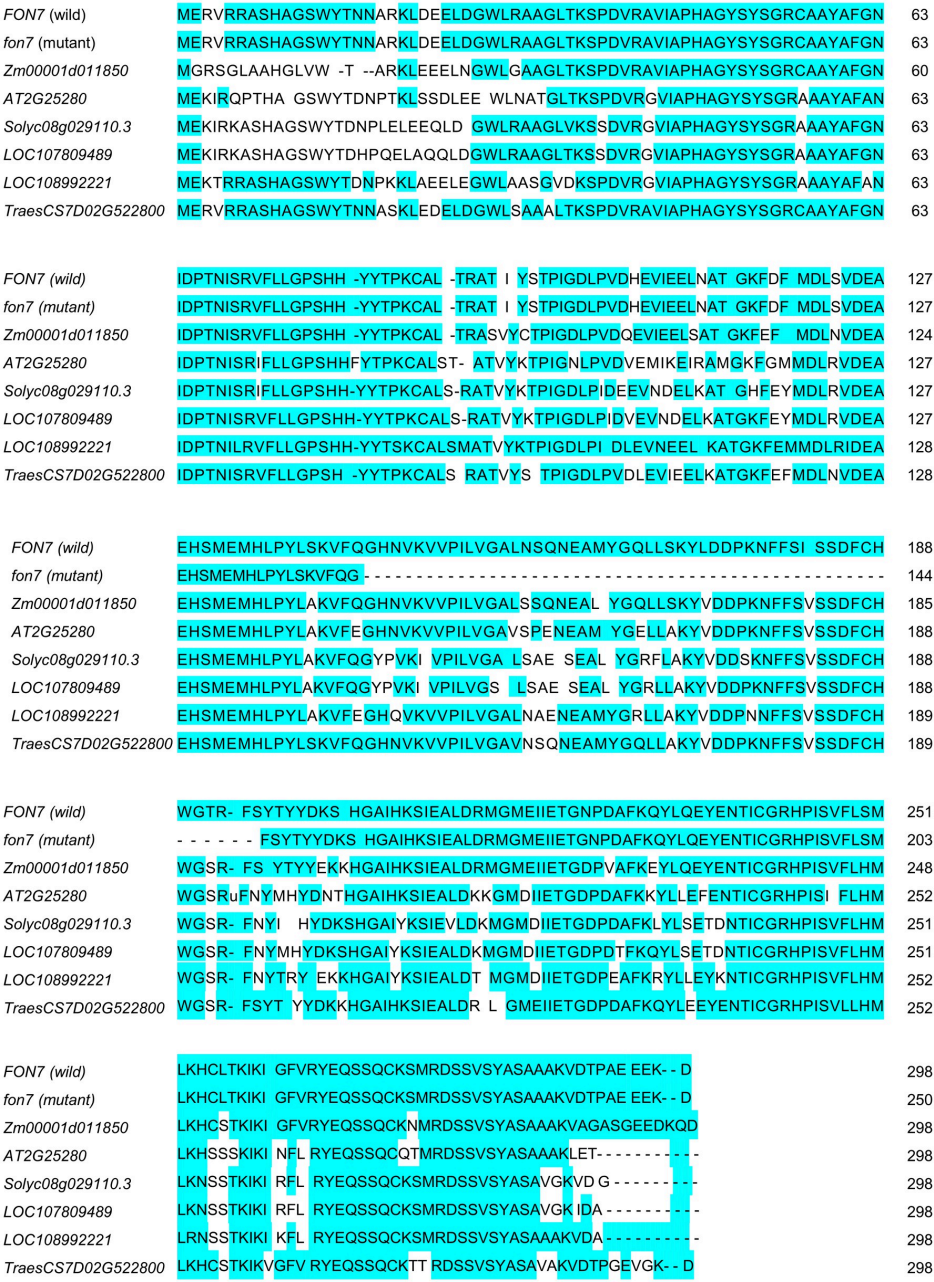
Multiple sequence alignment of MEMO1 homologs. Amino acid sequence alignment of MEMO1. Pale-blue box represents identical or similar amino acids. *Zm00001d011850*, *Zea mays*; *AT2G25280*, *Arabidopsis thaliana*; *Solyc08g029110*.*3*, *Solanum lycopersicum*; *LOC107809489*, *Nicotiana tabacum*; *TraesCS7D02G522800*, *Triticum aestivum*.

*FON7* (*Os08g0299000*) encodes a member of the mediator of ErbB2-driven cell motility protein (MEMO1) family. A total of 62 genes in 47 organisms, including human, animals, and plants, were found to encode MEMO1 proteins, 80% of which occurred in plant species. Multiple sequence alignment indicated that rice MEMO1 shared high sequence identity with its homologs in corn (*Zm00001doaa850*, 86%), wheat (*TraesCS7D02G522800*, 83%), tobacco (*LOC107809489*, 73%), tomato (*Solyc08g029110*.*3*, 72%), and *Arabidopsis* (*AT2G25280*, 69%) (Fig 6). The MEMO1 protein is a newly found plant signaling protein with unknown function. The signaling pathway of MEMO1 needs to be investigated in future studies.

## Discussion

Rice spikelets are the ultimate sink organs, and produce seeds as the final products. Female reproductive organs (pistils) and male reproductive organs (stamens) play crucial roles in the fertilization process, and the number of these organs affects success rate of seed setting and mature grain development [2]. In this study, we isolated a novel floral organ number regulatory gene, *FON7*, which was named after six genes (*FON1*, *FON2/FON4*, *FON3*, *FON5*, and *FON6*) previously reported to influence floral organ identity. The first abnormal floral organ number phenotype was reported in *multiple pistil-1* (*mp1*) and *mp2* mutants, which exhibit floral homeotic transformation [6]. Of the six genes previously reported to regulate floral organ number, only three genes (*FON1*, *FON2/FON4*, *FON3*) have been cloned and functionally characterized to date. While the approximate chromosomal positions of *FON5* and *FON6* have been determined, their exact gene-related information and biological functions remain unknown. The *fon1*, *fon2*, *fon5*, and *fon7* mutants were generated by chemical mutagenesis, whereas *fon3* was found to exist as a spontaneous mutant in the Gu-Guang-Huang farm line [17, 28–31]. The *fon5 and fon7* mutants exhibit similar phenotypes, i.e., increased stamen and pistil numbers and homeotic conversion of stamens and pistils (Fig 1j–1o) [31]. In *fon1* and *fon2* mutants, the lodicule number is altered, and homeotic conversion is limited to lodicules and stamens [29]. However, the *multi-grain 1* (*mg1*) mutant, a novel allele of *fon1*, shows an increasing number of stamens and pistils, extra lemma-like and extra palea-like organs, and changing spikelet meristem determinacy [32]. The *fon3* mutant exhibits strong homeotic conversion, which is characterized by the alteration of nearly all floral organ numbers and noticeable changes in panicle morphology [29].

The *fon7* mutant identified in the current study showed a considerable reduction in tiller number and plant height compared with the wild-type (Table 1 and S1 Fig). These phenotypes were similar to those of floral organ number mutants *fon1-3* and *fon1-4*, in which the reduced tiller number was caused by increased auxin production from the enlarged shoot apical meristem (SAM) and enhanced apical dominance [17]. Apical dominance is enhanced by the production of plant hormones such as auxin, which inhibit the growth of axillary buds, thus reducing the tiller number [33]. Among all *fon* mutants identified to date, only the *fon5* mutant exhibited the early flowering trait of *fon7* (Table 1) [31].

*FON1* encodes the CLV1 receptor kinase, and *FON2/FON4* encodes the CLV3 homolog of Arabidopsis. Thus, the *FON1* and *FON2/FON4* genes regulate floral organ number through the CLV–WUS signaling pathway. Our results indicated that *FON7* encodes the MEMO1 protein, which is also involved in the regulation of floral organ number [34]. A major outcome of MEMO1-mediated signaling is cell migration, which is an essential event during organismal development, adult homeostasis (e.g., cellular immunity, wound healing, etc.), and pathogenesis (e.g., tumor metastasis) [35]. MEMO was reported as an oxidase, and was shown to sustain NOX-mediated O_2_^-^ production and to increase localized ROS abundance [36]. MEMO1 contributes to the overall redox state of the cell through oxidize Ras Homolog Family Member A (Rho A), possibly interacting with other key redox regulators, and creates a localized oxidized environment conducive for signaling and migratory purposes [35, 36]. According to a recent study, Rho-like small G proteins such as RAC/ROPs act as switches of multi-functional signaling that affects leaf epidermal cell morphogenesis, polarized cell growth, and hormone [37]. In rice, OsRopGEF7B regulates floral organ development, and loss of OsRopGEF7B increases the number of floral organs in the inner whorl (stamen and ovary), leading to abnormal lemma and ectopic lodicule growth, and eventually reducing seed setting [38]. In this study, the loss of MEMO1 protein led to increased inner whorl floral organ number (stamen and ovary) and reduced floret fertility. Detailed functional analysis of the rice MEMO1 protein is needed to understand its role in floral organ number determination.

In the *fon7* mutant, 37% of the florets showed an increasing number of floral organs, including up to nine stamens and four stigmas. In addition, the *fon7* mutant showed the multiple-grain phenotype (Table 1). Multiple grains were not formed by all abnormal florets but only by florets containing four stigmas. The occurrence of multiple grains, also known as twin grain, multi-grain, and polycarpellary grain, has also been reported in the other *fon* mutants identified previously, including *fon1* (*mg1*), *fon2*/*fon4*, and *fon3*. Recently, the *twin grain1* (*tg1*) gene (allelic to *fon2/fon4)* was introgressed into the cytoplasmic male sterile (CMS) line, and a new CMS line was established with enhanced glume opening, stigma exsertion, high outcrossing rate, and high hybrid seed yield. Ye et al. (2017) suggested that floral organ number genes show great potential for increasing hybrid seed yield, and the floral organ mutant could serve as a valuable germplasm for CMS hybrid rice breeding [39]. This implies that the *fon7* mutant could be used to improve CMS lines for increasing the production of hybrid seeds.

## Conclusion

This investigation clearly shows that the *fon7* gene mutation increases floral organ numbers in the inner whorl (stamens and pistils), and leads to the formation of multiple grains. Identification of additional genes and MEMO proteins involved in floral organ number variation would help to elucidate the molecular mechanisms underlying floral organ development.

## Supporting information

S1 Fig Comparison of plant morphology between the wild-type and *fon7* mutant. (a, b) Plant morphology (a) and panicle morphology (b) of the wild-type and *fon7* mutant. (c) Morphology of internode length. (d) Comparison of internode length between the wild-type and *fon7* mutant.
(TIF)

S2 Fig Germination of two seeds from multiple-pistil grains. (TIF)

S3 Fig Application of MutMap to the F2 mapping population. Single nucleotide polymorphism (SNP)-index plots of 12 chromosomes generated by the MutMap analysis. The genomic region with the highest SNP-index peak harboring the candidate mutation is shown. Green and orange lines indicate 95% and 99% confidence intervals, respectively. Blue dots represent SNP-index values at the SNP position. Y-axis shows SNP-index values ranging from 0–1, and X-axis indicates the SNP position (Mb).
(TIF)

S1 Table Floral characteristics and plant morphology of the wild-type and *fon7* mutant. (XLSX)

S2 Table Mutations identified on all chromosome of rice plants with multiple pistils. (XLSX)

S3 Table Summary of SNPs identified on chromosome 8 of mutant rice plants with multiple pistils. (XLSX)

S4 Table Summary of candidate SNPs (SNP-index = ~1) on chromosome 8. (XLSX)

S5 Table Details of primers used in this study. (XLSX)
