# Supplementary figures and images for: Identification and characterization of a novel gene controlling floral organ number in rice (Oryza sativa L.)
Source: PLoS One. 2023 Jan 5;18(1):e0280022. doi: 10.1371/journal.pone.0280022 (PMC9815651; doi:10.1371/journal.pone.0280022)

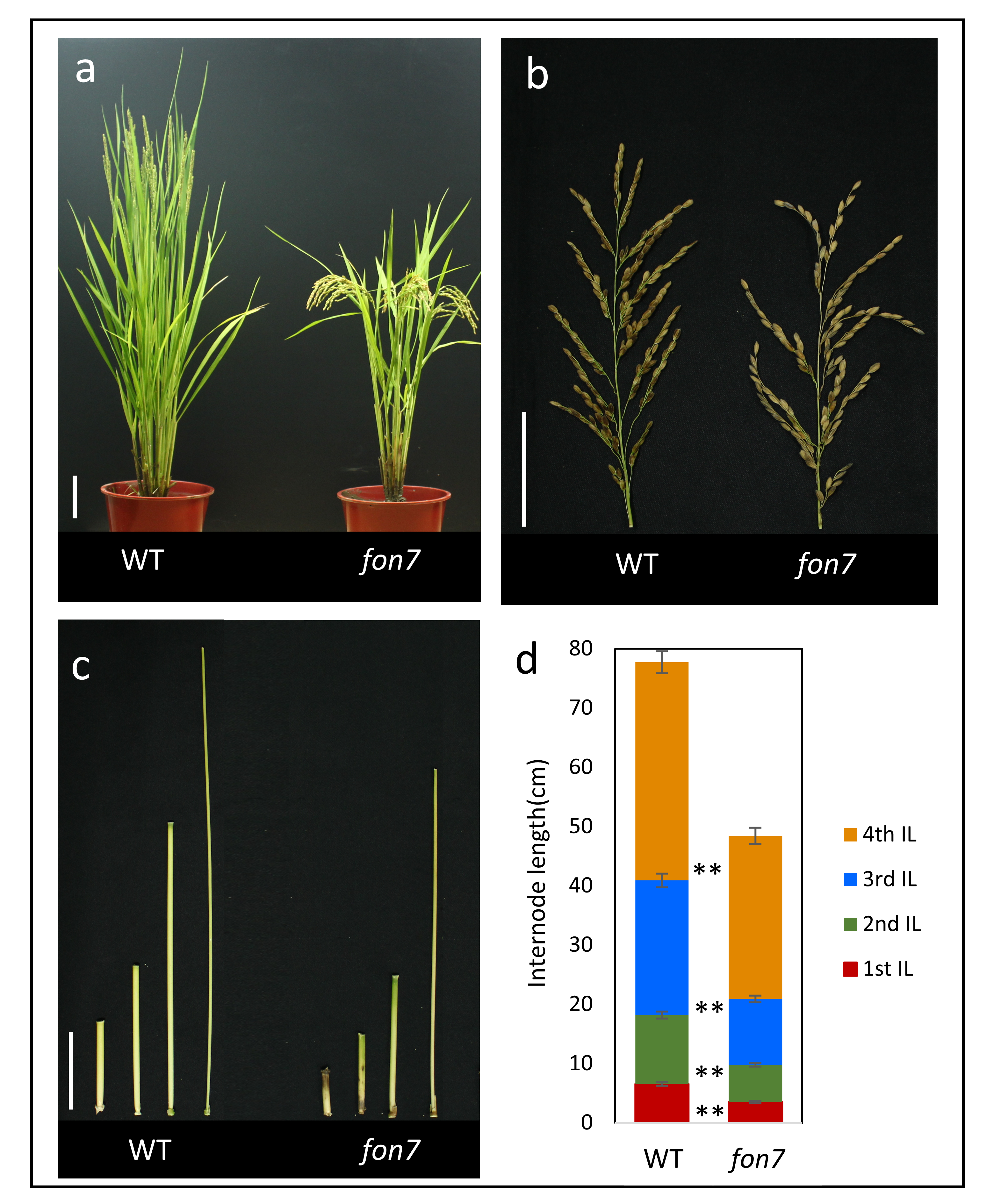

Supplement: S1 Fig — (a, b) Plant morphology (a) and panicle morphology (b) of the wild-type and fon7 mutant. (c) Morphology of internode length. (d) Comparison of internode length between the wild-type and fon7 mutant. (TIF) [file pone.0280022.s001.tif]

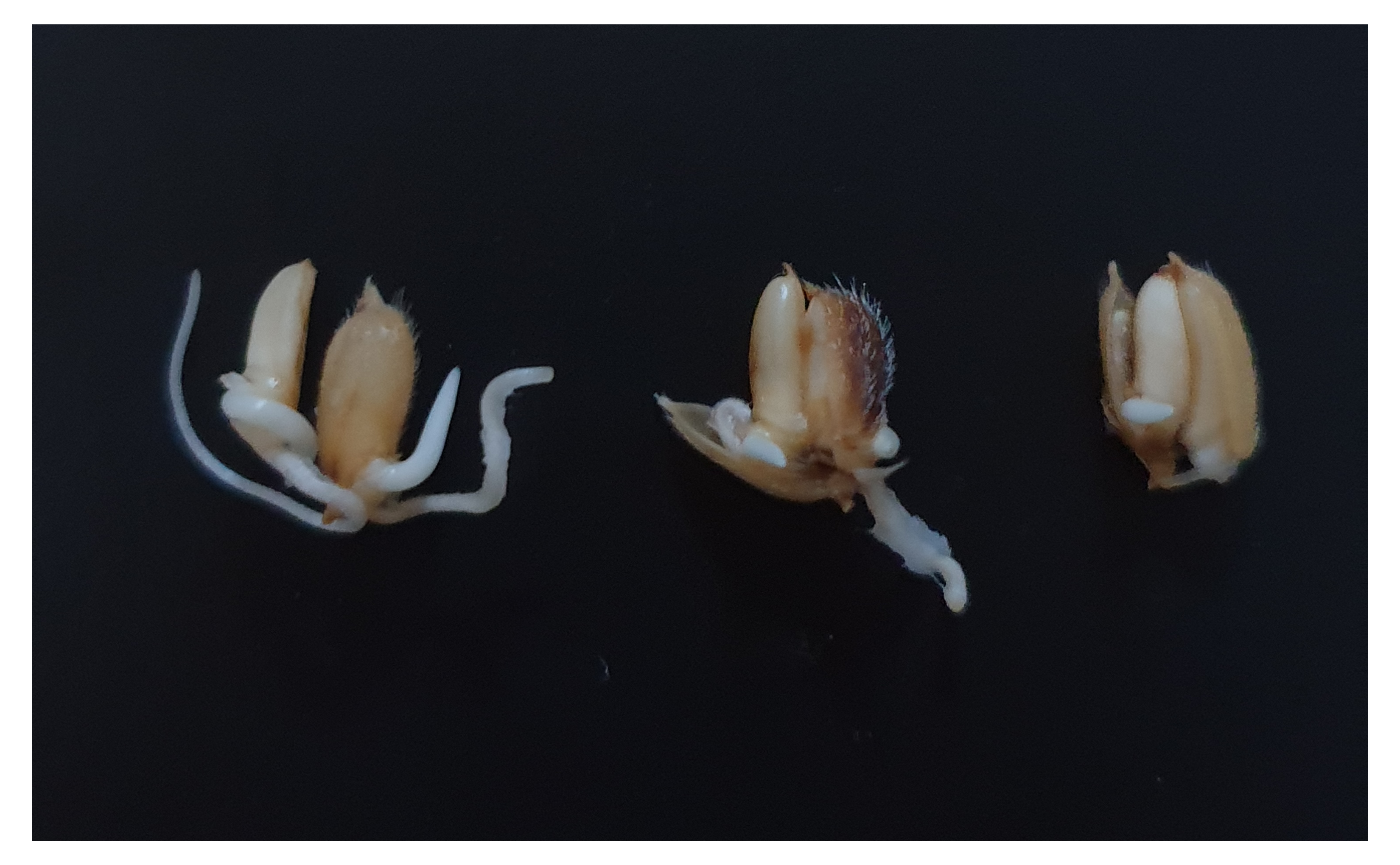

Supplement: S2 Fig — (TIF) [file pone.0280022.s002.tif]

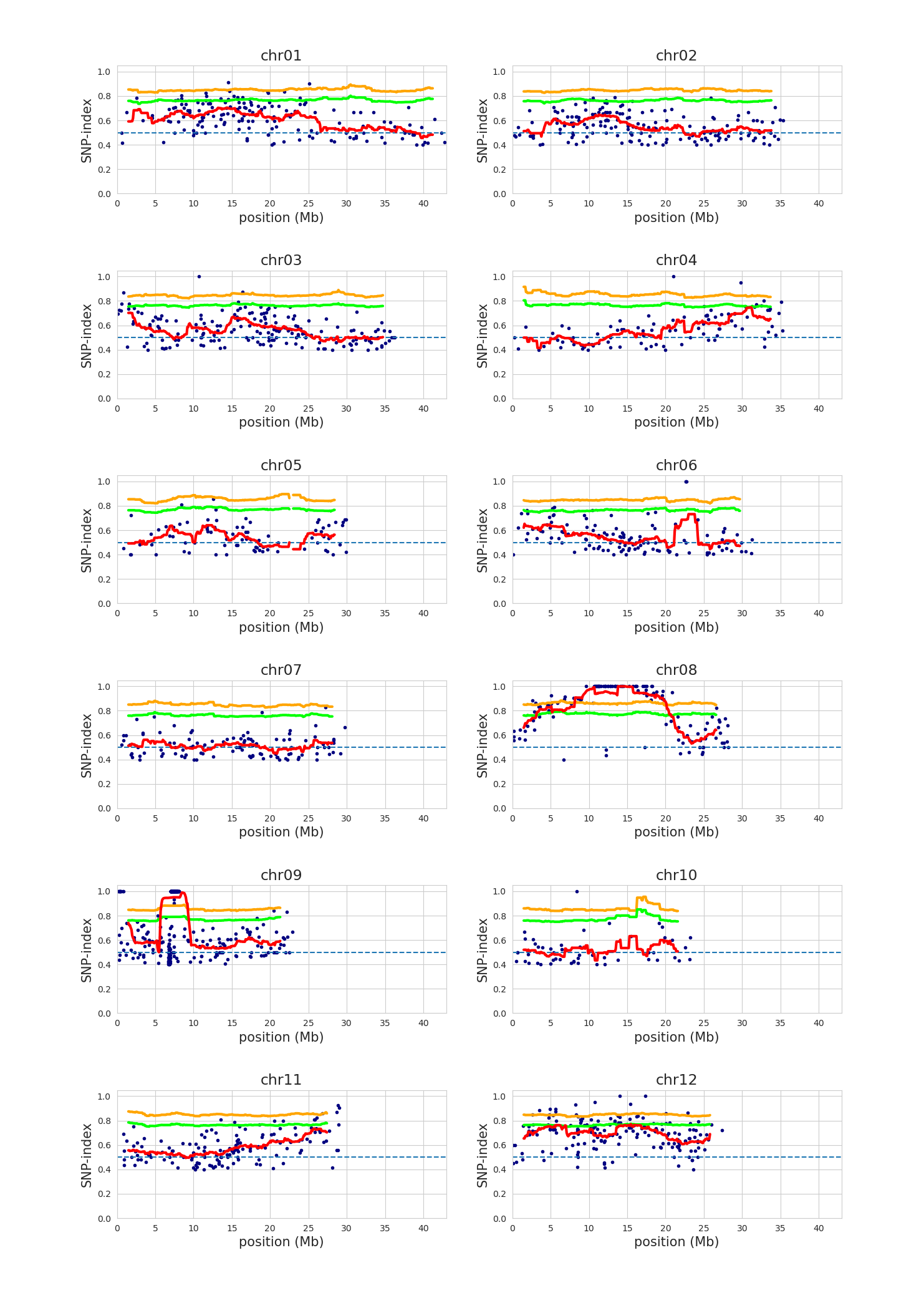

Supplement: S3 Fig — Single nucleotide polymorphism (SNP)-index plots of 12 chromosomes generated by the MutMap analysis. The genomic region with the highest SNP-index peak harboring the candidate mutation is shown. Green and orange lines indicate 95% and 99% confidence intervals, respectively. Blue dots represent SNP-index values at the SNP position. Y-axis shows SNP-index values ranging from 0–1, and X-axis indicates the SNP position (Mb). (TIF) [file pone.0280022.s003.tif]
